# The International Urogynecological Association/International Continence Society classification of complications of prosthesis and graft insertion: Pros and cons and a review of the literature

**DOI:** 10.4274/jtgga.galenos.2019.2019.0036

**Published:** 2020-03-06

**Authors:** Murat Yassa, Ozan Doğan

**Affiliations:** 1 Clinic of Obstetrics and Gynecology, Şişli Hamidiye Etfal Training and Research Hospital, İstanbul, Turkey

**Keywords:** Calculator, complications terminology, female pelvic floor surgeries, graft, prosthesis

## Abstract

International Urogynecological Association (IUGA) and the International Continence Society (ICS) and the Joint IUGA/ICS Working Group on Complications Terminology formulated a standardized terminology and classification of complications related to the use of prosthesis in female pelvic floor surgeries. It was mainly purposed to globally standardize the complications and related definitions in order to obtain factual rates and to enable comparisons and surgical audits. Although this unique classification has frequently been cited in the literature, some concerns have been raised against its complexity of use and inter- and intraobserver variability. This review aimed to discuss the rationale behind the IUGA/ICS complication classification system, underline the opposing views, and provide the Turkish version of an online calculator facilitating the universal coding to increase the utility.

## Introduction

The International Urogynecological Association (IUGA) and the International Continence Society (ICS) and the Joint IUGA/ICS Working Group on Complications Terminology formulated a standardized terminology and classification of complications related to the use of prosthesis in female pelvic floor surgeries (1). This classification system is the first attempt to systematically classify the related complications. It was mainly purposed to globally standardize the complications and related definitions in order to obtain factual rates and to enable comparisons and surgical audits. Although this unique classification currently has over 150 citations (https://citations.springer.com/item?doi=10.1007/s00192-010-1324-9, data received at 11/02/2019), some concerns has been raised against its complexity of use and inter- and intraobserver variability (2,3). This non-systematic review aimed to discuss the rationale behind the IUGA/ICS complication classification system, underline the opposing views, and provide the Turkish version of an online calculator facilitating the universal coding to increase the utility (Supplement).

## Rationale

Mid-urethral sling is the gold standard and the most common surgical procedure to treat stress urinary incontinence (SUI) with a proven superiority over other surgical procedures (4). Although mid-urethral slings exhibit a good safety and effectivity profile, a safety concern has been raised globally against the vaginal use of mesh, particularly to treat pelvic organ prolapse. The use of synthetic mesh has statistically decreased between 2011 and 2013 after the second United States Food and Drug Administration Public Health Notification; however, the number of mesh revision surgeries increased by almost three-fold from 2007 to 2013 (5).

A recent meta-analysis consisting of 28 randomized controlled trials (RCTs) and 15,855 patients showed that patients who received mid-urethral sling had higher overall and objective cure rates than those who underwent Burch colposuspension (4). The latest Cochrane systematic review assessing mid-urethral slings for SUI determined that major complications such as nerve, bowel or major vascular injuries, pelvic haematoma, necrotizing fasciitis, ischiorectal abscess, and death were found to be uncommon in mid-urethral slings (6). Bladder perforation, reoperation, urinary retention, pelvic haematoma, infection, vaginal tape erosion/extrusion and groin pain occurred in 3.9%, 2.4%, 1.6%, 1.9%, 0.7%, 1.5% and 0.4% of women underwent to retropubic tape procedure, respectively. Those rates were 0.4%, 2.2%, 0.5%, 0.5%, 0.6%, 0.4%, and 1.6% for transobturator tapes (6). Another large population-based retrospective series consisting of 95,057 women was recently published (7). Women who had their first mid-urethral sling procedure to treat SUI were included and followed for 5.5 (interquartile range, 3.2-7.5) years. They found that the rate of mesh sling removal was 1.4% at 1 year, 2.7% at 5 years, and 3.3% at 9 years. The rate of all reoperations was found as 2.6%, 5.5%, and 6.9%, at 1, 5, and 9 years, respectively. By contrast, the recent largest study of vaginal mesh in the treatment of SUI including 92,246 women, revealed that almost one out of every ten patients experienced a complication within 5 years of the initial mesh surgery (8). Among those, rate of complications have risen during the surgery and in the first month were found as 2.4% and 1.7%, respectively.

It has been argued that RCTs designed for long-term follow-up possess limited information about whether there was a hidden cache of serious adverse effects that might have been set against the benefits of curing incontinence (6). Many reporting systems belonging to the major registries were characterized by passive surveillance systems limited by the inclusion of the potential submission of incomplete or inaccurate data, under-reporting of events, lack of denominator data, and the lack of report timeliness ) (6).

Due to the inconsistent and increasing reports of complication rates, the IUGA and ICS proposed a well-detailed but inclusive classification system of complications related to the use of all types of prostheses including meshes, implants, tapes, and grafts in female pelvic floor surgery (1).

## Classification system and coding

**Figure 1:**
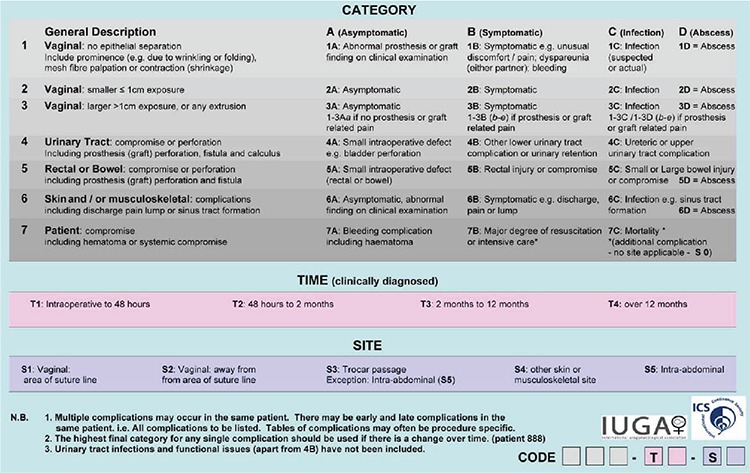
The IUGA/ICS classification system of complications of prosthesis and graft insertion^1^ IUGA: International Urogynecological Association, ICS: International Continence Society

The IUGA/ICS system was developed to cover all possible physical complications, including trocar-related insertion complications and healing abnormalities. The classification system depends on three main factors: Category (numeric) and division (letter), Time (numeric + letter), and Site (numeric + letter), respectively, and all together, this is called the cheque truncation system (CTS). “Category” refers to the general description of the complication such as the degree or extent of erosion (according to former usage), affected site or the condition of the patient. “Division” refers to four common major complication types: A-Asymptomatic, B-Symptomatic, C-Infection, D-Abscess. “Time” describes the duration between the surgery and clinically diagnosed complication. “Site” describes the localization of the complication. One can obtain a code of 3 letters and 3 numerals after classification (e.g. 2B/T3/S1) (Figure 1). The only sub-group reflects “pain” according to the vaginal examination and/or anamnesis. Pain adds a lower case next to the division (e.g. 2Bc/T3/S1, if a patient expresses pain during sexual intercourse).

One of the main prominent features in the newly proposed joint terminology is that the term erosion is not favoured. Mesh inherently interacts with adjacent tissue. Therefore, it is replaced by terms of vaginal epithelium separation, exposure, extrusion, contraction, prominence, and sinus tract formation. Additional new terms include compromise, perforation, and dehiscence (1). Although exposure can simply be described as visible or palpable mesh through separated epithelium (mainly the vaginal wall) in the early period, extrusion represents a subsequent delayed process by which mesh protrudes gradually out of a body structure or adjacent tissue such as the vagina, bladder, and urethra. Perforation frequently refers to perioperative events. In addition, the classification system has dynamic characteristics. Naturally, multiple complications may occur in the same patient at the same time or over a period of time and all should be reported separately (1).

The boundaries of the CTS system include not covering the urinary tract infections, functional issues (e.g. voiding dysfunction), intraperitoneal adhesions and prion or viral infection of a xenograft. Secondly, recurrence is not situated in the CTS system because recurrence is not counted as a complication. Those exclusions are probably postulated to be not directly related to the insertion of prosthesis. Lastly, complications linked to the bulking agents are also not included.

## Literature and opposing views

Petri and Ashok assessed the applicability of the IUGA-ICS classification by retrospectively analysing 359 patients who underwent surgical management due to a complication directly related to insertion of a synthetic sling and classified each complication according to the new IUGA-ICS classification using an online calculator (https://www.ics.org/complication). Although they found that the new classification system had good general applicability, it was inadequate to classify overactive bladder (OAB), which was accounted as the most common complication with a rate of 54% (n=193). Lower urinary tract obstruction requiring resection or cutting the sling was the second most common complication at 48% (n=174). This complication was classified as 4B; however, the authors could not state the “Site”. Except those two, the CTS system was beneficial in classifying most of the rare and common complications. Along with including OAB and sub-classifying 4B, Petri and Ashok also recommended some other rare complications to be labelled as miscellaneous such as dyspareunia of the partner, urine loss during intercourse, and foreign body sensation in the vagina.

In 2015, Miklos et al. (9) analyzed mesh complications among women who had undergone pelvic floor reconstructive surgery with mesh including sub-urethral mesh slings, transvaginal synthetic mesh, and sacrocolpopexy in their multi-centre retrospective study. A total of 445 patients were included from three tertiary urogynecological referral centers. Unlike Petri and Ashok, all of the complications that mainly consisted of complicated and often recurrent cases were possible to be classified using the IUGA-ICS classification system in their study.

Tunitsky et al. (2) retrospectively analyzed 1236 patients and identified 133 eligible patients presenting after pelvic organ prolapse or incontinence surgery with 195 mesh-related complications in their study to assess the interrater reliability of the IUGA-ICS classification. The complications were classified by 2 independent reviewers using the ICS/IUGA classification system. They observed low agreement at 44.09% on vaginal complications (categories of 1A-3D), high agreement on urologic (96.1%, categories of 4A-4C) and bowel complications (100%, categories of 5A-5C). The authors claimed that 2.2% of the complications could not be classified into any organ/severity categories, and the “Site” of the complications could not be located in 38% due to the lack of clarity of the IUGA-ICS classification. Interestingly, they also observed low agreement on “complication time” and “complication site” between the two independent reviewers with 47.6% and 29.7%, respectively. Tunitsky et al. (2) suggested that complications might be classified by symptom and intervention rather than the physical findings. For example, they argued that Category 5, which was designated for bowel complications, did not cover defecatory dysfunction. Although that proposal would probably increase the complexity of the classification system, we believe that Tunitsky et al. (2) might have a point, particularly in pelvic organ prolapse surgeries, but not necessarily in anti-incontinence procedures (10,11). We were able to explain all complications using the CTS system after insertion of old- and new-generation mid-urethral slings to treat USI.

The feasibility and the difference of the complication system between prolapse and anti-incontinence surgeries was assessed in a single-centre retrospective study that used a wide range of surgical kits (12). The most frequent complications varied with the type of the surgery, which were found to be bladder outlet obstruction for vaginal sling-plasty, and pain for prolapse surgery. The affected site also differed between them, but the time remained statistically similar. The authors commented that using the CTS code might provide a quick overview of patients’ major findings in a more general way; however, the complication classification system needed to evolve in a such way that it covered functional disorders (e.g. urgency, constipation, and dyschezia) given that 17.32% (n=31/179) of the patients presented with only functional problems in their study.

Following the assertion of poor interrater reliability of the IUGA-ICS classification, Gowda et al. (3) had similar results in their study that stratified interobserver reliability by stage of training. It should be noted that the authors stated their study was underpowered and had sampling bias. As a response to studies showing poor interrater and interobserver reliability, the original authors ran the hypothesis that the poor reproducibility was because of imperfect study designs and that the reliability could be strengthened through optimized training prior to use of the CTS IGAU/ICS complication classification system. Haylen et al. (13) achieved excellent interobserver reliability (93%) with no significant differences among 39 respondents after giving structured instructions supported by photos and quizzes, even though the participants were under time pressure and had no access to the online calculator.

Batalden et al. (14) assessed the retrospective applicability of the IUGA/ICS classification system. The authors only included complications with mesh erosion and the newly expanded definitions consisting of contraction, prominence, separation, exposure, extrusion, perforation, dehiscence, and sinus tract formation. They observed that the classification did not predict the treatment or outcome of the complication, and 30% of the mesh erosions could not be retrospectively coded with the CTS system. However, it was mainly due to missing information that did not exist in the clinical documentation or operative reports.

Bontje et al. (15) specifically assessed the complications of patients who consecutively underwent vaginal prolapse repair using mesh. The authors were able to code 43 complication from 39 patients out of 107 (36.45%). They stated that the classification system was found to be generally successful, but only needed to expand the coverage such as the need of reoperation, the duration of the impact of the complication, and severity of bleeding. In a small scaled retrospective study with 57 patients, Hammett et al. (16) drew attention to the rate of the resolution of symptoms after mesh removal. They showed that the complete resolution or improvement rate was 57.3% with the use of the IUGA/ICS classification system.

## Conclusion

The IUGA/ICS complication classification system is one of a kind and the first universal classification coding system facilitating the standardized data accumulation and surgical audit specifically for vaginal prostheses. The system can be enriched and strengthened by covering urinary functional problems. Although gaining a prompt and deep insight into the CTS system seems difficult, the online calculator can accurately simplify classification.  We believe that the complications’ classification system should be increasingly used to achieve an objective and international agreement. This may allow to standardize documentation, leading to a more accurate assessment of complications and their severity.
